# Predicting self-perceived general health status using machine learning: an external exposome study

**DOI:** 10.1186/s12889-023-15962-8

**Published:** 2023-05-31

**Authors:** Jurriaan Hoekstra, Esther S. Lenssen, Albert Wong, Bette Loef, Gerrie-Cor M. Herber, Hendriek C. Boshuizen, Maciek Strak, W. M. Monique Verschuren, Nicole A. H. Janssen

**Affiliations:** 1 National Institute for Public Health and the Environment (RIVM), Bilthoven, The Netherlands; 2 Institute for Risk Assessment Sciences, Utrecht University, Utrecht, The Netherlands; 3 Wageningen University & Research, Wageningen, The Netherlands; 4 Julius Center for Health Sciences and Primary Care, University Medical Center Utrecht, Utrecht University, Utrecht, The Netherlands

**Keywords:** Exposome, Machine learning, Random forest, Self-perceived general health

## Abstract

**Background:**

Self-perceived general health (SPGH) is a general health indicator commonly used in epidemiological research and is associated with a wide range of exposures from different domains. However, most studies on SPGH only investigated a limited set of exposures and did not take the entire external exposome into account. We aimed to develop predictive models for SPGH based on exposome datasets using machine learning techniques and identify the most important predictors of poor SPGH status.

**Methods:**

Random forest (RF) was used on two datasets based on personal characteristics from the 2012 and 2016 editions of the Dutch national health survey, enriched with environmental and neighborhood characteristics. Model performance was determined using the area under the curve (AUC) score. The most important predictors were identified using a variable importance procedure and individual effects of exposures using partial dependence and accumulated local effect plots. The final 2012 dataset contained information on 199,840 individuals and 81 variables, whereas the final 2016 dataset had 244,557 individuals with 91 variables.

**Results:**

Our RF models had overall good predictive performance (2012: AUC = 0.864 (CI: 0.852–0.876); 2016: AUC = 0.890 (CI: 0.883–0.896)) and the most important predictors were “Control of own life”, “Physical activity”, “Loneliness” and “Making ends meet”. Subjects who felt insufficiently in control of their own life, scored high on the De Jong-Gierveld loneliness scale or had difficulty in making ends meet were more likely to have poor SPGH status, whereas increased physical activity per week reduced the probability of poor SPGH. We observed associations between some neighborhood and environmental characteristics, but these variables did not contribute to the overall predictive strength of the models.

**Conclusions:**

This study identified that within an external exposome dataset, the most important predictors for SPGH status are related to mental wellbeing, physical exercise, loneliness, and financial status.

**Supplementary Information:**

The online version contains supplementary material available at 10.1186/s12889-023-15962-8.

## Background

Self-perceived general health (SPGH) is a comprehensive and sensitive indicator of an individual’s health status commonly used in epidemiological research [1]. It is determined using a simple question such as “In general, would you say that your health is…”. Poor SPGH is a strong predictor for hospitalization and mortality [1, 2] and the ability to accurately predict SPGH status would therefore create valuable insight in the development of SPGH. A wide range of exposures belonging to different domains are known to be associated with SPGH status, such as (health-related) lifestyle [3], neighborhood environment [4], distance to urban green spaces [5], air pollution levels [5], and several biomarkers [6]. The majority of these studies were performed using multivariate regression models based on a limited number of predictors. Also, most assumed linear relationships between exposures and SPGH status, with limited interactions between the different predictors. Ideally, models for predicting SPGH status should be based on the entire multifactorial exposure of an individual and allow for interaction between these exposures.

To take this full multi-environmental exposure on the development of epidemiology into account, the concept of the exposome was developed [7]. Expanding on the early concept proposed by Dr. Wild [8], the definition of the exposome was refined by Miller & Jones as the “cumulative measure of environmental influences and associated biological responses throughout the lifespan, including exposures from the environment, behavior, diet, and endogenous processes” [9]. The exposures that make up the exposome are generally divided into three overlapping domains: internal, specific external and general external exposures [8]. The internal domain includes biological factors such as oxidative stress, gut microflora and metabolism rate [8], whereases the specific external domain represents individual-specific exposures such as lifestyle, diet, occupation, and emotional state. These are traditionally obtained through survey and questionnaire data. The general external domain includes predominantly exposures based on the area of residence of an individual, such as socioeconomic status of neighborhood, noise, and distance to urban green spaces [8].

Although an exposome study approach can benefit epidemiological research, there are some practical drawbacks. By definition, exposome studies use a large number of different exposures. Traditional (logistic) regression models used in epidemiological research are less suitable in these situations since these regression models have difficulty in capturing complex relationships between (co-) variables, and dealing with high dimensional data, especially when highly correlated variables are involved. Therefore, the usage of machine learning (ML) techniques might be a valid alternative for regression models in epidemiological exposome studies and a suitable (non-linear) ML technique for prediction / classification tasks is random forest (RF) [10, 11]. This ensemble learning method is based on the construction of multiple, independent decision trees which each use different subsets of the data [11]. A comparative study on the performance of RF and logistic regression models demonstrated that in most situations, RF models perform better in binary classification tasks than logistic regression. This superiority of RF was more pronounced in datasets with an increasing number of variables [10]. The usage of RF is well established in other research fields than epidemiology and its application in epidemiology research, while still limited [11], shows promising results [12]. Although RF models have been criticized for being hard to interpret, there are several techniques available to create more insight in the models beyond just performance metrics such as accuracy, model error and the area under the curve (AUC) of the receiver operating characteristic (ROC) curve. A variable importance (VI) procedure allows for identification of the most important individual predictors within the RF model [13]. In addition, the (non-linear) relationships between individual exposures and the outcome variable within the RF can be estimated using partial dependence (PD) and accumulated local effect (ALE) plots [13].

The aim of this study was to develop prediction models for SPGH status based on large external exposome datasets using the non-linear ML technique RF and identify the most important predictors for SPGH status.

## Methods

### Study population

The study populations were based on the respondents of the 2012 and 2016 editions of a 4-yearly national health survey (Public Health Monitor Adults and Elderly of the Community Health Services, Statistics Netherlands and the National Institute for Public Health and the Environment, 2012 and 2016, *Gezondheidsmonitor Volwassenen en Ouderen 2012 en 2016, GGD’en, CBS en RIVM).* This public health monitor (PHM) included questions on personal and lifestyle characteristics, socioeconomic status, and physical, mental, and general health. This cross-sectional survey is conducted every four years in The Netherlands by regional Public Health Services (GGD), Statistics Netherlands (CBS) and the National Institute for Public Health and the Environment (RIVM). By design of the PHM, people aged 65 and above were oversampled. The 2012 edition of the survey included information on 387,195 individuals and the 2016 edition on 457,153 individuals. Only a limited number of individuals participated in both editions of the survey (*n* = 17,500). Two separate datasets were created based on these two editions of this survey, allowing for comparison of the model results.

### Personal characteristics

An overview of all personal characteristics is given in Additional file 1 and are here further introduced. Standard socio-demographic characteristics, such as gender, age, country of birth, education level, household composition, and marital status were all available from the PHM. The respondents were also asked for information on their weight and length, based on which their body mass index (BMI) was calculated. The survey also scored general, emotional, and social loneliness using the De Jong-Gierveld loneliness scale. In brief, individuals replied to six different statements on loneliness, of which three were indicators of emotional and three were indicators of social loneliness, and based on their answers were assigned a score for general, emotional, and social loneliness [14]. Furthermore, several lifestyle-related characteristics (alcohol consumer status, number of alcoholic consumptions per week, smoking status, minutes of physical exercise per week) were also included. In addition to questions on gainful employment, unemployment and being incapacitated, the PHM also contained the question “In general, does it take effort to make ends meet*”* with the possible answers “none” / “almost none”/ “some” / “large”. This question informs if respondents had a high enough monthly income to provide for their basic needs.

The 2016 edition of the PHM contained additional questions regarding if respondents were active as volunteers and if they had a feeling of being in control over their own life, with the possible answers being *“*inadequate*” / “*moderate*” / “*much*”*. Furthermore, information was also available on if respondents met the physical activity guidelines for balance (at least once per week perform balance exercises), mobility (at least 150 min of light physical activity per week, spread over at least 2 days) and muscle- and bone-strengthening exercises (at least twice per week) [15].

### Neighborhood characteristics

For each respondent, the PHM was enriched with their geocoded residential address and based on this we linked several neighborhood characteristics from 2012 and 2016 from the StatLine database of Statistics Netherlands [16, 17]*.* For each characteristic, values at a neighborhood level were used. This level is the most detailed geographic unit of neighborhood characteristics available, and one unit represents an average of approximately 600 households [16, 17]. Multiple neighborhood sociodemographic and socioeconomic characteristics were linked for each participant, such as the percentage of inhabitants with a non-western migration background, the percentage of inhabitants with a western migration background, percentage of residents between age 15–64 and above 64, percentage of rental housing and average property value in the neighborhood [16, 17]. The density of addresses per Km^2^ in the neighborhood was also included in the study, both as the absolute number of addresses and divided into categories using the standard definitions of urbanicity of Statistics Netherlands (“very highly urban” ≥ 2,500 addresses per Km^2^; “highly urban” = 1,500–2,500 addresses per Km^2^; “moderately urban” = 1,000–1,500 addresses per Km^2^; “few urban” = 500–1,000 addresses per Km^2^; “not urban” < 500 addresses per Km^2^) [16, 17]. In addition to address density, the absolute number of inhabitants, average income of inhabitants, number of households, average household size, and the average numbers of cars per household were also included. Furthermore, we used information on the relative number of different types of governmental benefits (unemployment, social and financial support), births and deaths per 1000 inhabitants in the neighborhood. Ease of access to health institutions (hospital, GP) and schools (daycare, schools, large schools) measured by the average distance (Km) calculated by road were also added [16, 17]. Besides these socioeconomic variables, we also included data on the amount of water surface area in hectares, the average gas (in m3) and electricity-use (KwH) in the neighborhood [16, 17]. Finally, data on the relative number of different types of criminal activities (burglaries, vandalism cases and violence & sexual crimes assaults) in the neighborhood was used in the study but was only available for 2016 [17]. An overview of all neighborhood characteristics is given in Supplementary Table 1, Additional file 1.

In addition to these characteristics from the StatLine database, information on the specific public health services region in which the respondent lived was also included. Due to internal reorganizations of the Dutch public health services, the number of unique regions differed between the 2012 (28 regions) and 2016 PHM (25 regions).

Certain neighborhood characteristics are neighborhood averages of characteristics that are also available at the individual level. For example, both each participant's reported income and the average income within each participant's neighborhood were used as exposures in the final dataset. Both types of exposures were used in this study because they represent different types of exposures (e.g. personal wealth vs living in a low-income neighborhood) that could influence SPGH status.

### Environmental characteristics

The long-term average concentrations of particle matter (PM10, PM2.5) and NO_2_ from the Dutch National Air Quality Cooperation Program (NSL) on the residential addresses of each subject was used. When information on this level was not available, the nearest value to the address within a 10-m buffer zone was selected using X, Y coordinates. Since NSL data was not available every year, concentrations of 2010 were used for the 2012 dataset [18, 19]. In addition, exposure to PM_coarse_, PM_2.5abs_ and two oxidate potential (OP) measurements of PM (electron spin resonance (OP^ESR^) and dithiothreitol (OP^DTT^)) at the residential address of each subject were assessed as described by Klompmaker et al. [5]. Furthermore, the 24-h daily average (Lden) of road-, airplane- and rail-traffic noise the on the residential address was also linked [20].

Residential surrounding green was assessed in buffers with radii of 300 m and 1000 m for the residential address of each respondent using the Normalized difference vegetation index (NDVI) and a highly detailed national land-use database of the Netherlands of 2010, as described by Klompmaker et al. [5]. Land-use data was divided into four separate factors: total surrounding green (excluding private green property and street greenery) (gre), urban (urb), natural (nat), and agricultural green space (agr). Since land-use data was not available across the Dutch border, no value could be attributed to a residential address within these radii. In addition, residential addresses of PHM individuals were also enriched with a selection of other environmental exposures. Information on the 10-year average number of animal species (Biodiversity score) and the number of red list endangered animal species per Km^2^ (Red list biodiversity score) was obtained from the biodiversity maps of Atlas Natural Capital, which are based on the data of the Dutch National Databank Flora and Fauna [21]. The biodiversity map from 2017 was used to assign biodiversity values for both datasets. The summer-averaged daily urban heat island (UHI) effect and the green space cooling (GSC) effect were obtained from the heat island maps of the Atlas Natural Capital based on modeled data from the Dutch National Institute for Public Health and the Environment [22]. For both the 2012 and 2016 datasets, the UHI and GSC values from the 2017 map were used. Indicator scores for mobility-friendliness of residential neighborhoods (Kernindicator beweegvriendelijke omgeving, (KBO)) were collected from maps of Atlas Living Environment based on data from the Mulier institute [23]. The KBO indicator is a score on a 1–5 scale, which indicates how much the local environment allows and supports mobility and an active lifestyle based on the number of sporting facilities, playgrounds, bicycle roads and recreational blue and green areas [23]. This indicator was only available for usage within the 2016 dataset.

### Outcome variable

The following question from the PHM was used to define a subject’s SPGH status: “In general, would you say that your health is….” with possible answers being “very poor” / “poor” / “moderate”/ “good” / “very good”. The response was dichotomized and individuals who answered “poor” or “very poor” were considered as individuals with poor SPGH.

### Random forest models

All analysis was performed similarly for both datasets using the R statistical software v4.0.2 [24]. The RF algorithm was implemented using the ranger package [25]. The number of trees within the RF model was fixed at 1000 and the tuning parameter “mtry”, representing the number of variables that are randomly sampled as candidates at each split, was optimized using the caret [26] package. For both datasets, the analysis was performed on the complete cases of each dataset. We assessed the performance of the RF models by five-fold cross validation (CV) and the ROC and AUC metrics were determined using the roc package [27]. The AUC values are presented as the arithmetic mean of the AUC of each cross-validated model, together with their 95% CI. The optimal sensitivity and specificity of each RF model are also presented as the mean of the optimal sensitivity and specificity values of the cross-validated models.

### Variable importance rankings, partial dependence and accumulated local effect plots

The VI rankings of the optimized models were determined by a permutation-based approach using the iml package [13]. Briefly, for each variable an RF model was made in which the values of the variable of interest were randomized. Next we compared the prediction performance of this model to the performance of the original model using the out-of-bag (OOB) error. The OOB measurement is used to estimate the prediction error of RF models [28]. By looking at the difference between the OOB error of the randomized model and the original model, the relative importance of each variable was determined [13]. This procedure results in a ranking, listing the most important predictors within the overall model. The VI value is expressed as the absolute difference between the OOB error of the permutated model and the original error. For each RF model, the VI procedure was repeated three times and the average score is presented.

To validate the VI rankings, the AUC value of RF models with a gradual increasing number of included variables was also assessed. The order in which variables were added was based on the VI ranking of the complete model. This validation step identifies which variables are required to build a RF model with optimal prediction performance using the least amount of included exposures [29]. Due to computational reasons, only the first 30 variables of the VI ranking were used in this analysis.

For a selection of variables with a high VI score, partial dependance (PD) and accumulated local effect (ALE) plots were created using the pdp [30] and the iml packages [13]. The PD procedure measures the effect of a variable on the outcome prediction of an RF model by marginalizing all other variables and estimating the relationship between the outcome probability and the values of the variable of interest [13]. The ALE estimates the conditional contribution of each variable by calculating the average differences in predictions. This blocks the effect of correlated variables on the prediction outcome, increasing the reliability of ALE plots when dealing with correlated data [31]. Due to technical reasons related to unevenly distributed categorical variables, the effects of categorical exposures were estimated using PD plots and effects of continuous exposures by ALE plots. As predictions from ALE plots became unreliable when based on a limited number of observations, only values between the 5th-95th percentile of the respective variable are presented. All results were visualized using the ggplot2 package [32].

### Selection of variables

Highly correlated variables can influence the outcome and interpretation of the VI ranking of RF models [33] and therefore variables with a Pearson’s correlation coefficient of more than 0.90 or less than -0.90 were not used together within exposome dataset. When two (or more) variables were highly correlated, one variable was removed. The decision which variable was to be excluded was based on 1) its availability within the 2012 and 2016 dataset, 2) its relevancy in the context of possible health effects and 3) the outcome of a VI ranking of a RF performed with all variables. A variable that was available in both years was preferred over variables that were only present one dataset. If there was no difference in availability nor in relevancy, the variable with the highest value in the VI ranking was selected for the final dataset. This resulted in two datasets with 81 (2012 dataset) and 91 unique variables (2016 dataset), from which 81 variables were present in both datasets. The final study populations were based on the complete cases of these datasets.

## Results

### Population descriptives and model prediction performance

The final study population within the 2012 dataset consisted of 199,840 respondents and the study population in the 2016 dataset contained information on 244,557 respondents. There were 7,081 (3.5%) respondents with poor SPGH in the 2012 population and 10,031 (4.1%) in the 2016 population. A full overview of the descriptives of the two study populations and an correlation matrix of all continuous exposures of the 2016 study population are given in Additional files 2, 3, 4, 5 and 6.

**Fig. 1:**
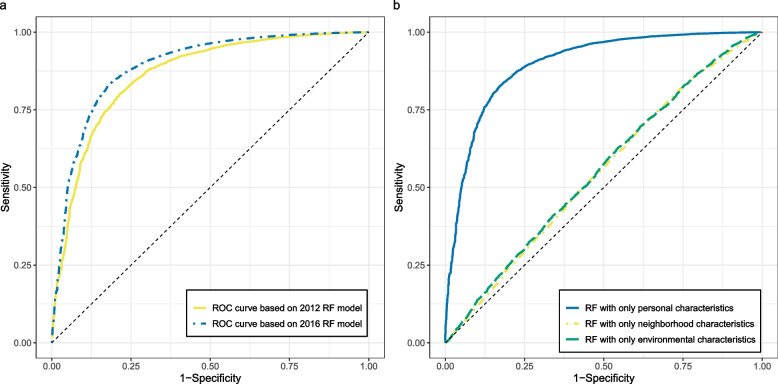
Receiver operator characteristic curves (ROC) for random forest models predicting self-perceived general health. Legend: The left panel **a** displays ROC curves of models based on the entire 2012 and 2016 datasets, whereas the right panel **b** shows the ROC curves of model based only on three different types of characteristics (personal, neighborhood and environmental) within the 2016 dataset. Displayed curves are based on the model with the median area under the curve (AUC) value out of the fivefold cross-validated models. The dashed line indicates a ROC with an AUC of 0.5

Performance metrics of the RF models to predict SPGH status in the different study populations are presented in Table 1. Receiver operator curves estimating the discrimination of the models predicting SPGH status yielded an AUC of 0.863 (CI: 0.851 – 0.876) for the 2012 RF model and an AUC of 0.890 (CI: 0.885—0.895) for 2016 (Fig. 1a). To assess the contribution of the different types of characteristics to the model performance, we used multiple combinations of variables within RF models. For both the 2012 and 2016 dataset, RF models based exclusively on personal characteristics performed strongly (AUC > 0.870), surpassing the prediction performance of the models containing all available variables (Table 2, Fig. 1b). Models containing only environmental characteristics (AUC < 0.573), or neighborhood characteristics had weak predictive strength (AUC < 0.564) (Table 2, Fig. 1b).

**Table 1:** Performance metrics of random forest models

	**2012 dataset**	**2016 dataset**
N	199,840	244,557
N poor SPGH status	7,081	10,031
N variables	81	91
MTry	9	9
N trees	1000	1000
AUC^a^ (95% CI)	0.864 (0.852– 0.876)	0.890 (0.885—0.895)
Sensitivity^b^ (95% CI)	0.777 (0.753 – 0.800)	0.811 (0.789 – 0.833)
Specificity^c^ (95% CI)	0.818 (0.793 – 0.842)	0.837 (0.820 – 0.854)

^a^Average AUC of five-fold crossed validated random forest models

^b^Average of five-fold crossed validated random forest models

^c^Average specificity of five-fold crossed validated random forest models

**Table 2:** Performance metrics of random forest models based on combinations of personal, neighborhood and environmental characteristics

Exposure Types	2012 Dataset		2016 Model Dataset	
	**N variables**	**AUC**^**d**^** (95% CI)**	**N variables**	**AUC (95% CI)**
**Personal** **Characteristics**^**a**^	29	0.875 (0.862 – 0.888)	34	0.898 (0.894 – 0.902)
**Neighborhood** **Characteristics**^**b**^	23	0.563 (0.544 – 0.582)	25	0.561 (0.553 – 0.569)
**Environmental** **characteristics**^**c**^	29	0.572 (0.556 – 0.588)	32	0.550 (0.540 – 0.558)
**All**	81	0.864 (0.852– 0.876)	91	0.890 (0.885—0.895)

^a^Variables from the Public Health Monitor on sociodemographic characteristics, lifestyle, loneliness, and financial status

^b^Air pollution, noise levels, distance to urban green, area biodiversity levels and neighborhood water surface area variables

^c^Neighborhood variables on social benefits, high- and low-income households, neighborhood demographics etc.

^d^Mean AUC of five-fold crossed validated random forest models

### Variable importance rankings

**Fig. 2:**
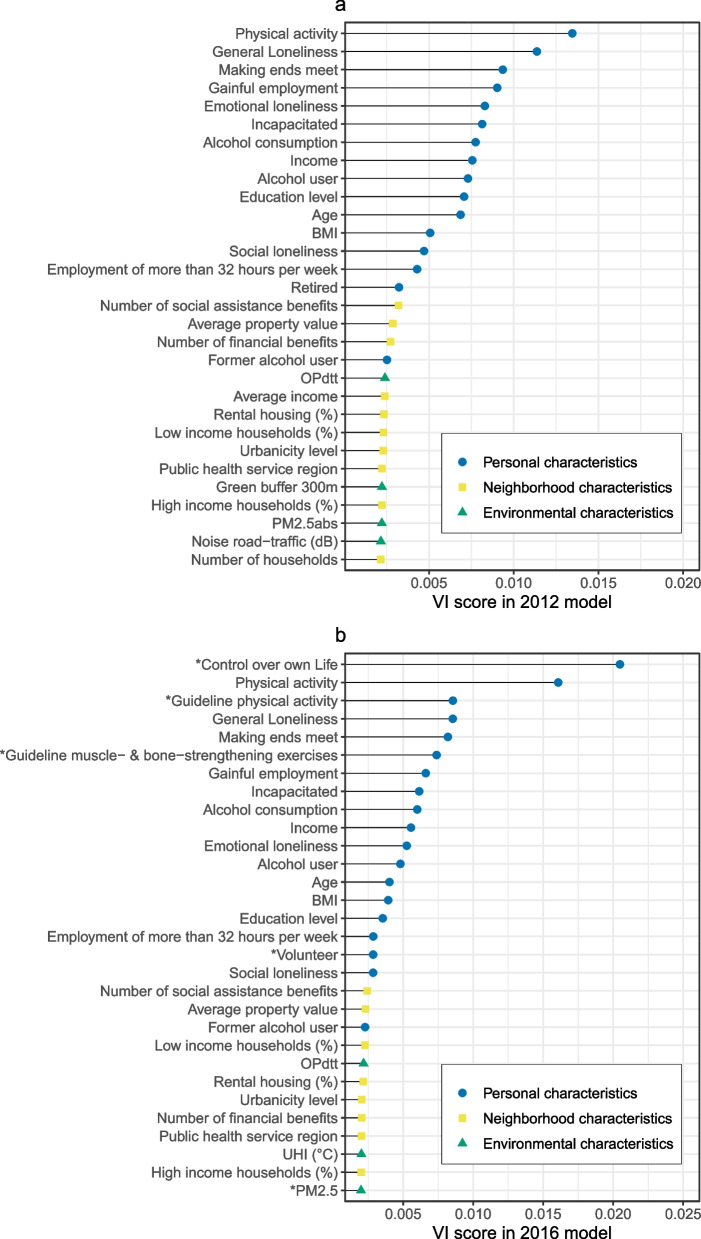
Top 30 variables from the variable importance rankings of RF models based on the 2012 and 2016 datasets. Legend: The left panel **a** shows the variable importance (VI) rankings of RF models performed on the entire 2012 dataset (n = 199,840, variables *n* = 81) and the right panel **b** the ranking based on the 2016 dataset (*n* = 244,557, variables *n* = 91). The VI is expressed as the difference between the original error of the RF model and the error after randomization of the exposures of interest. The VI procedure was performed in triplicate, and the average VI score is presented. Point shape represents the type of variable: personal characteristics, neighborhood characteristics and environmental characteristic. Variables listed with an * were only available within the 2016 dataset

The 30 variables with the highest VI score based on the different RF models are presented in Fig. 2 and an overview of the entire ranking is given in Additional file 7. Within the 2012 RF, the five most important variables were “Physical activity”, “General Loneliness”, “Making ends meet”, “Gainful employment” and “Emotional loneliness” (Fig. 2a) The five variables with the highest VI score in the 2016 RF model were “Control of own life”, “Physical activity “, “Guideline physical activity”, “Making ends meet” and “General loneliness” (Fig. 2b). Multiple top variables of the VI ranking of the 2016 model were only available within this dataset, such as “Control of own life” (rank nr 1), “Guideline physical activity” (rank nr 3) and “Guideline muscle- and bone-strengthening exercise” (rank nr 6).

**Fig. 3:**
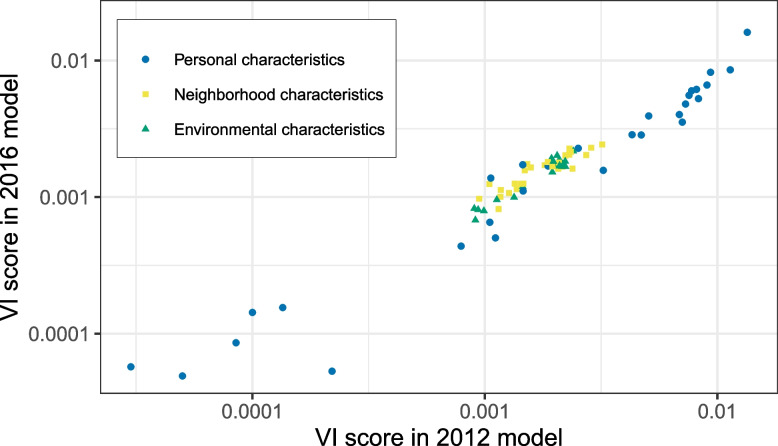
Relationship between variable importance (VI) score of variables present in both the 2012 and 2016 RF model. Legend: The VI score expressed as the difference between the original error of the model and the error after randomization of the variable of interest. The VI procedure was performed using the iml package in R in triplicate, and the average score is presented. Point color represents the type of variable: personal characteristics, neighborhood characteristics and environmental characteristic

Personal characteristics were the overall most important type of exposures within the models, making up the first 15 and 18 variables in the 2012 and 2016 rankings, respectively. The variable “Social assistance benefits in neighborhood” was the highest-ranking neighborhood characteristic in both models (rank nr 16 in 2012 and rank nr 19 in 2016), followed by “Average property value” (rank nr 17 in 2012 and rank nr 20 in 2016). Among the environmental exposures, the “OP^dtt^” variable ranked highest in both the 2012 (rank nr 20) and 2016 (rank nr 23) models. There was an overall strong correlation between the VI score within the 2012 and the 2016 RF models (Pearson’s *r* = 0.946, 95% CI = 0.917—0.964) among the variables (*n* = 81) that were present in both datasets (Fig. 3).

**Fig. 4:**
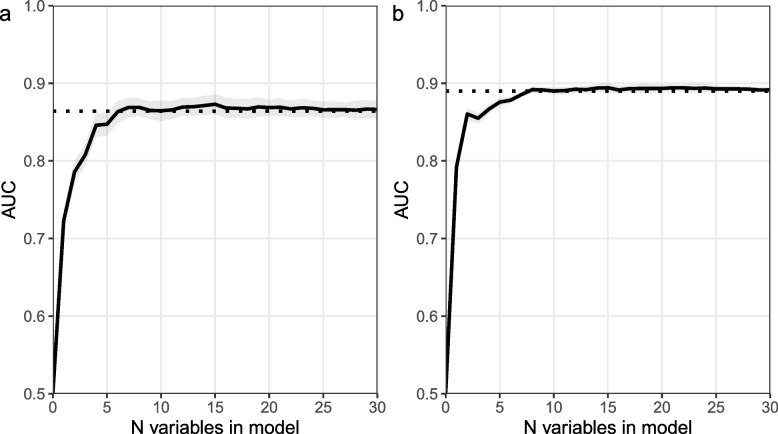
Effect of increasing the number of variables within RF models on the AUC value. Legend: Left panel **a** shows results from models based on the 2012 dataset and right panel **b** of models based on thee2016 dataset. The AUC values represent the average AUC based on fivefold cross validation and its 95% CI in RF models with an increasing number of variables inside the RF models. Default settings of the ranger package were used in the assembly of each RF model. Variables are added in an order that corresponds to the variable importance ranking of the complete model. The dashed line represents the average AUC value of the RF forest containing all variables (2012: *n* = 81; 2016: *n* = 91)

Next, the AUC values of RF models with a gradually increasing number of variables based on the VI ranking were determined. Including the first seven variables of the VI ranking within a RF gives a model with similar performance as the model containing all variables from the 2012 dataset (Fig. 4a). For the 2016 dataset, a model containing the top eight variables of the ranking performed equally to the full RF model (Fig. 4b).

### Top predictors of self-perceived general health status

**Fig. 5:**
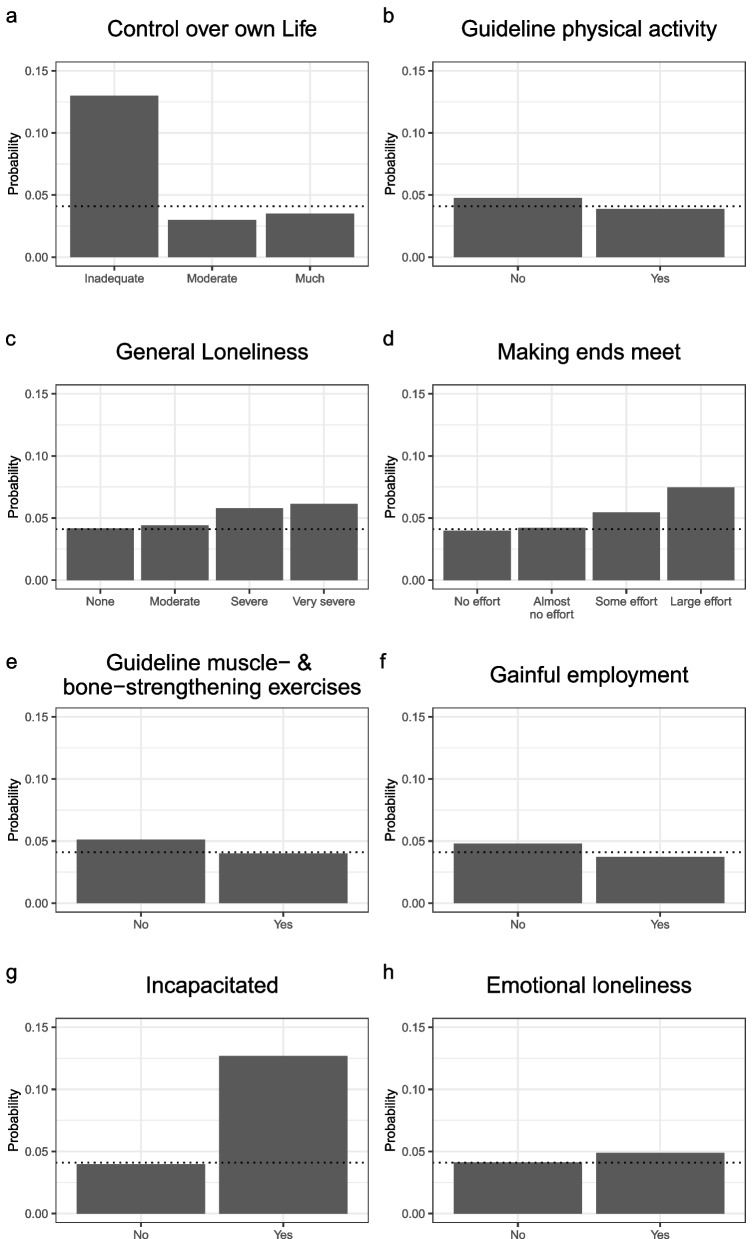
Partial dependance plots of a selection of top ranked categorical variables within a random forest model to predict self-perceived general health status. Legend: The following variables from the complete 2016 random forest model (*n* = 244,557, variables *n* = 91) are presented: **a** “Control of own life” (VI rank: 1), **b** “Guideline physical activity (VI rank: 3), **c** “General loneliness” (VI rank: 4), **d** “Making ends meet” (VI rank: 5), **e** “Guideline muscle- and bone-strengthening exercise” (VI rank: 6), **f** “Gainful Employment” (VI rank: 7), **g** “Incapacitated” (VI rank: 8), and **h** “Emotional loneliness” (VI rank: 11). Dashed line represents the probability of poor SPGH status in the dataset without taking any exposures into account (0. 041)

The top categorical variables within the RF models were explored using PD plots. Since the importance ranking and PD/ALE plots of variables of both RF models were highly similar, only the results of the 2016 model are presented. A feeling of inadequate control over one's own life increases the probability of poor SPGH, whereas a feeling of moderate or much control was associated with good SPGH (Fig. 5a). Respondents who did not match the guidelines for physical activity or muscle- and bone-strengthening exercise had an increased probability of poor SPGH (Fig. 5b, e). Having no loneliness or a moderate loneliness score did not influence an respondents’ SPGH status, but a severe or very severe loneliness score increased the probability of poor SPGH (Fig. 5c). Furthermore, effort with making ends meet also increased the probability of having poor SPGH, and the probability of poor SPGH was higher for respondents with a large effort compared to respondents with some effort (Fig. 5d). Being unemployment or incapacitated also increased the likelihood of poor SPGH (Fig. 5f-g). Finally, respondents who scored as being emotionally lonely had a higher probability of poor SPGH (Fig. 5h).

**Fig. 6:**
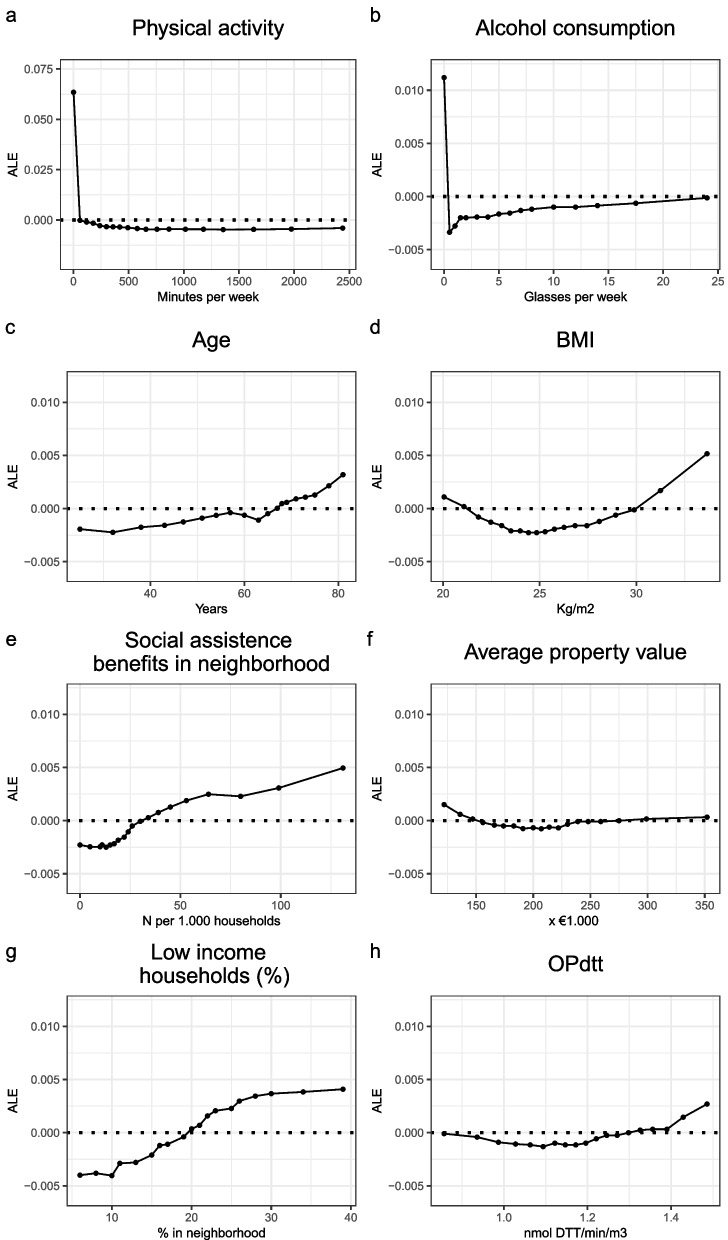
Accumulated local effects plots of a selection of top continuous variables within a random forest model to predict self-perceived general health status. Legend: The following variables from the complete 2016 random forest model (*n* = 244,557, variables *n* = 91) are presented: “Physical activity” (**a**, VI rank: 2), “Alcohol consumption” (**b**, VI rank: 9), “Age” (**c**, VI rank: 13), “BMI” (**d**, VI rank: 14), “Social assistance benefits in neighborhood” (e, VI rank: 19), “Average property value” (**f**, VI rank: 20), “Low income households” (**g**, VI rank: 22) and “OPdtt” (**h**, VI rank: 23). Points represent the percentile rank in steps of 5 percentile points, starting from the 5th and ending with 95th percentile of population. The dashed line represents the (normalized) average prediction of the entire RF model

The associations of individual continuous exposures within the RF models were investigated using ALE plots. Being physically inactive greatly elevates the probability of poor SPGH and increased physical activity per week lowers this probability but this protective effect plateaus at 750 min per week (Fig. 6a). Persons who consume no alcoholic drinks during the week have an increased probability of poor SPGH. However, low-level consumption of alcoholic drinks has a protective effect on SPGH, which decreases gradually with increased levels of consumption (Fig. 6b). In addition, the likelihood of poor SPGH status increased with age (Fig. 6c) and persons with either a BMI below 21 or above 30 also had a higher probability of poor SPGH status (Fig. 6d). Among the most important neighborhood characteristics, the probability of poor SPGH increased with the number of social assistance benefits in the neighborhood (Fig. 6e). A low average property value in the neighborhood also increased the chances of poor SPGH, but the ALE plot also indicated an increased probability of poor SPGH in neighborhoods with the highest property values (Fig. 6f). However, the effect size of low average property values was stronger than that of high property values. Furthermore, the likelihood of poor SPGH status increased with percentages of low-income households living in the neighborhood (Fig. 6g). The ALE plot of the most important environmental variable revealed that persons with exposed to PM with a high OP value (> 1.3 nmol DTT/min/m3) have an increased probability of poor SPGH status (Fig. 6h).

Additional PD and ALE plots of variables within the top 30 best predictors of SPGH status in the 2016 RF model are presented in Figures S1 and S2, Additional file 8.

## Discussion

In the current study, we developed RF predictive models for SPGH status based on two different external exposome datasets and identified the strongest predictors of SPGH status within these models. Overall, the predictive accuracy of our models was strong, achieving AUC ranging between 0.864 and 0.890. The most important predictors in these models were all personal characteristics related to mental wellbeing, physical exercise, loneliness, employment, and financial status.

The current study was conducted within the framework of the exposome, in which exposures are defined being internal, specific external or general external exposures [9]. However, there is considerable overlap between these three domains of the exposome and it may be difficult to classify specific exposures into one domain or another. This is particularly true for exposures related to physical activity, stress and emotional states, as these can be considered part of both the internal and specific external domains [34]. However, in the context of the exposome, the term "internal exposure" refers primarily to internal biological processes measured by high-throughput molecular techniques in metabolomics, proteomics and transcriptomics [35]. Although the datasets used in this study include some exposures that could be considered internal emotional states (e.g. loneliness, feeling of control), we did not examine any internal biological markers and therefore consider our study an external exposome study.

In this study, the SPGH is used as a measure of an individual's health status. The advantages of the SPGH are that it is easy to implement in studies, it captures many dimensions of health and it has been identified as a predictor of mortality and other health outcomes [1, 2]. However, there are also limitations to the use of SPGH as an outcome. The measurement is based on personal experience of health problems/impairments and reference points, which can vary widely between individuals based on their demographic, socioeconomic or cultural background [36, 37]. Nevertheless, comparisons between SPGH studies have shown that although the prevalence of reported SPGH status may vary between countries, the associations of SPGH with covariates are highly consistent [38].

We identified the relative importance of variables within the RF models using a permutation-based approach. Although several studies investigated associations between different types of exposures and SPGH status [4, 5, 39], a novel element of our study is that it provides a direct comparison and ranking of the predictive performances of different types of predictors of SPGH status. In addition, the inclusion of more than 80 different variables from different domains also gives the opportunity to show the effects of individual exposures within a complex interactive model. The “Control of own life” variable was the strongest predictor within the RF models based on the 2016 dataset (not available for 2012) and persons with a feeling of no control were more likely to have poor SPGH. This association between feeling in control of one's own life and several health outcomes has previously been demonstrated using logistic regression models [40]. Possible explanations for the relationship between SPGH status and a feeling of control are the known associations between a feeling of low control over one’s life and several health risk behaviors [41, 42], which can contribute to poor SPGH status. Due to the cross-sectional nature of this study, feeling not in control could also be a direct consequence of a person’s health problems. The “physical activity” variable was another important predictor of SPGH status, and ALE plots based on this exposure show that increased amounts of physical activity per week reduces the probability of poor SPGH. This outcome agrees with several other studies that also link a lack of physical activity with poor SPGH [39, 43]. This is not surprising since the positive effect of physical activity on general and mental health is well described in literature [44, 45]. The probable underlying biological mechanisms of this effect include enhanced neuroplasticity and growth factor expression, promoting an anti-inflammatory state of the body and optimizing the endocrine and physiological response to physical and psychological stress [46]. However, it is also likely that some individuals within the study population have low levels of physical activity as a direct result on their poor health status. Other top predictors for SPGH included the ability to make ends meet, gainful employment and loneliness (both general and emotional), which have all been previously linked with SPGH status [47, 48]. Possible explanations for the relationship between financial status and health status are the concepts of health selection or social causation. The health selection model proposes that poor health has a negative effect on educational and occupational attainment and consequently financial status. In contrast, the social causation model posits that social conditions, such as financial problems or bad housing conditions, are a trigger for problems with health [49]. The effect of loneliness on general health can be explained by the model of Hawkley and Cacioppo, which proposes that loneliness is accompanied by feelings of stress and anxiety, and this could result in a dispositional tendency which can influence genetic, neurological, or immune functioning [50]. The ALE plot of the predictor alcohol consumption shows an interesting relationship between SPGH and alcohol consumption since participants who didn’t consume any alcohol had the highest probability of poor SPGH. In addition, individuals with 0.5 drinks per week had the lowest probability of poor SPGH and the likelihood of poor SPGH increased with the number of drinks. This U-shaped relationship has also been observed in other studies [51, 52] and is probably due to the cross-sectional nature of survey studies, as health impairments are likely to have a major impact on drinking patterns due to lifestyle changes and medication [51].

The most important neighborhood variable in both datasets was “Social assistance benefits in neighborhood” and the ALE plot of this variable revealed a positive association between the standardized number of social assistance benefits and poor SPGH status. A similar association is seen for the percentage of low-income households in neighborhood exposure. High numbers of social assistance benefits to inhabitants and low-income households in the neighborhood are considered indicators of socioeconomically disadvantaged neighborhoods and living in these areas has previously been associated with poor SPGH status [4, 53]. The relationship between average property value and SPGH appears to be U-shaped, as both people living in an area with low (< 15th percentile) or high (> 90th percentile) property value have an increased risk of poor SPGH. However, the effect size (as estimated by the ALE) of living in a neighborhood with low-value housing is considerably stronger than that of living in a neighborhood with high property values. This skewed U-shaped relationship between SPGH and neighborhood economic status is also observed in the ALE plots for the variables rental housing (%) and high income households (%) (Additional file 8, Figure S2), suggesting a non-linear relationship between neighborhood wealth and SPGH status.

The highest-ranking environmental variables in both RF models was OP^dtt^*,* which ranked lower than the three highest neighborhood variables. The OP^dtt^ variable is a measurement of the oxidative potential for ambient PM using the DTT assay. Oxidative stress in cells induced by their interaction with air particles is considered one of the underlying mechanisms of PM-associated health effects, and the OP^dtt^ exposure thus incorporates biologically relevant properties of PM [54]. The ALE of OP^dtt^ indicates an increased probability of poor SPGH for individuals who are exposed to PM with high OP (> 70^th^ percentile). The previous study of Klompmaker et al*.* also showed that the OPdtt variable had the strongest association with poor SPGH compared to a set of other air pollution exposures [5].

While the previously discussed associations between SPGH status and a selection of environmental and neighborhood characteristics agree with multiple other studies which used logistic regression models [5, 55, 56], these characteristics generally did not rank high in the VI ranking of our RF models. A possible explanation for these low ranks is that these variables are associated with but not highly predictive of SPGH status, especially when compared to personal characteristics. Where logistic regression models identify associations between variables and (health) outcome based on a series of hypothesis tests and their resulting p-values, the RF models attempt to do so through the perceived contribution of each variable to the overall prediction performance. As seen in Fig. 4, only a handful of variables are required to construct a RF with similar predictive strength as the complete models. This by no means implies that other variables which do not (substantially) improve the prediction performance of the model are not associated with or influence a subject’s SPGH status, even if it is more likely that such variables are not associated. The ALE plots of high-ranking but not essential variables, such as for “Age”, “BMI”, “Social assistance benefits in neighborhood” and “OP^dtt^” do indicate a relationship between exposure and outcome. (Figs. 4  and 5). It is likely that these associations are too weak to accurately predict a subject’s SPGH status to add any predictive strength to the model in addition to the signal within the strongest predictors from the VI ranking. A possible explanation for this could be the fact that due to the cross-sectionally design of the used survey, which includes individuals throughout the entirety of The Netherlands, there are relatively many low exposed individuals for certain environmental (PM10, NO^2^, UHI) or neighborhood (Social assistance benefits, average housing price) exposures. This, in combination with an expected small effect size at low-level exposures, can contribute to why no major effects of these characteristics are observed and their low place in the VI ranking. The implications of this result on environmental policy are not fully clear. The outcomes of the RF suggest that at the most commonly observed exposure levels within our study population, environmental and neighborhood exposures do not have a major impact on general health status. If we assume that the observed associations between SPGH and neighborhood / environmental characteristics are causal in nature, lowering these exposures to the lowest observed value will only have a major impact on a small group of subjects in the study. However, it also possible that lowering these exposures will have a small positive health effect on a large part of the population and still result in a considerable overall impact on health, but this cannot be determined based on the current study. Future investigations should therefore further examine the relative impact of different types of external exposures on general health.

The focus of this study was on identifying the most important predictors of SPGH status and not investigating associations between all types of exposures and SPGH. Therefore, the evaluation of the relationship between all individual exposures and SPGH was outside our scope. Nevertheless, our results do indicate that care should be taken in the interpretation of the VI ranking of RF models that combine personal and environmental variables in epidemiological studies. Using a RF together with a VI procedure can however be useful as a pre-analysis in epidemiological studies to identify environmental exposures with the strongest effect on a health outcome of interest from a larger exposome dataset, which can then further be investigated using techniques more suitable for studying associations with an expected modest contribution to prediction performance, such as (logistic) regression models. A recent study by Ohanyan et al*.* used an approach in which a specific set of personal variables labeled as confounders (such as age, gender, employment status) were used within a RF model but excluded from the overall VI ranking to identify to most important environmental predictors in the study [57]. Although this is a straightforward approach, this does raise the question on how to decide which exposures to consider as a co-variable/confounder and which as a predictor. An alternative study design would involve performing an RF on a subset of data to identify the most important confounders / predictors of a health outcome. These confounders could then be used in a regression or permutation analysis on the remaining data to investigate the relationship between the environmental exposure of interest and the health outcome. Despite these considerations for the interpretation and practical applications of RF models, the usage of this technique still has major potential in the field of (environmental) epidemiology for its ability to handle large exposome datasets and its data-driven approach to identify key predictors of health outcomes and describe non-linear associations.

The datasets used in this study were based on two editions of the same public health survey, enriched for both years with environmental and neighborhood information. Despite minor differences in the survey questions and the available additional information available for each year, most of the variables were identical between the two datasets. There were minor performance differences between the two models, with the 2016 model performing slightly better. This is most likely explained by the availability of additional personal variables within the 2016 dataset, such as “Control of own life”, “Guideline physical exercise” and “Guideline muscle- and bone-strengthening exercise”. Differences in the number of cases of poor SPGH and age distribution between the two datasets are also possible explanations for the minor differences in performance of the models. Despite these performance differences, there was a strong correlation between the VI score among variables that were present in both datasets. This high similarity in the outcomes of the VI procedure performed on two, independent datasets is a strong proof of the robustness of the RF procedure and the outcomes of this study.

Strengths of the current study include a large sample (*n* > 432,000, divided over 2 datasets) and variable (*n* > 90) size. In addition, the direct comparison of the outcomes of models based on two independent datasets and the ranking of the relative predictive strength of SPGH predictors are novel elements that, to our knowledge, have not been previously performed. Finally, we used non-linear techniques (RF combined with ALE plots) to describe the relationship between exposure and SPGH status. We also recognize that this study has several limitations. First, due to the observational nature of this study we are unable to establish a causal relationship between exposure and SPGH status. It is likely that certain exposures (those related to physical activity and employment) could also be a result of a subject’s SPGH status. For instance, the ALE plot of the variable “Alcohol consumption” indicates that individuals who do not consume any alcohol are more likely to have poor SPGH, which is most likely a consequence of poor general health on drinking patterns. Secondly, the inclusion of correlated variables within the datasets might have influenced our study results. We tried to reduce the possible bias within the VI ranking caused by correlated variables by not including variables that had a higher correlation coefficient than 0.9 or -0.9. Despite these precautions, it is still possible that correlation between variables influenced the VI score of some predictors [33]. This would especially be true for correlated variables with an expected small effect on SPGH status, such as air pollution levels and neighborhood characteristics. Finally, it should be noted that the PHM, on which the datasets used in this study are based, is not a true random sample of the Dutch population. Due to the design of the survey, the elderly population (older than 64 years) is over-represented in the study population and therefore the results of this study cannot be directly generalized to the entire population of the Netherlands or other countries.

## Conclusions

This study identified that the most important predictors for SPGH status are related to mental wellbeing, physical exercise, loneliness, and financial status. Although associations between SPGH status and neighborhood / environmental characteristics were observed, they scored low on the VI ranking and were weak predictors of SPGH status within the RF model.

## Supplementary Information

**Additional file 1: Table S1.** Overview of variables used in RF models.

**Additional file 2: Table S2.** Descriptives of continuous variables in the 2012 dataset (*n* = 199,840)

**Additional file 3: Table S3.** Descriptives of categorical exposures of 2012 dataset (*n* = 199,840).

**Additional file 4: Table S4.** Descriptives of continuous variables of 2016 dataset (*n* = 244,557).

**Additional file 5: Table S5.** Descriptives of categorical exposures of 2016 dataset (*n* = 244,557).

**Additional file 6: Table S6.** Pearson r^2^ correlation matrix of numerical exposures of the 2016 dataset (*n* = 244,557)

**Additional file 7: Table S7.** VI rankings of RF models based on the 2012 and 2016 datasets.

**Additional file 8: Figure S1.** Partial dependance plots of a selection of top ranked categorical variables within a random forest model to predict self-perceived general health status. **Figure S2.** Accumulated local effects plots of a selection of top continuous variables within a random forest model to predict self-perceived general health status.

## Data Availability

The datasets used in this study were constructed based on multiple existing datasets from the Dutch public health services and Statistics Netherlands (public health monitor, neighborhood statistics), RIVM (air pollution, urban green buffers, noise levels) and NSL (air pollution). Restrictions apply to the availability of the PHM data, which were used under license for the current study. The final datasets are therefore not publicly available in an online repository. Requests to access the PHM data should be directed at the Dutch public health services (monitorgezondheid@ggdghor.nl). After permission from the Dutch public health services, the complete datasets used in this study can be made available by the corresponding author upon reasonable request. Access to the public data sources used in the study are provided with the paper. The following public domain resources were used: Statistics Netherlands, District and neighborhoods map 2016. Accessible from: https://www.cbs.nl/nl-nl/dossier/nederland-regionaal/geografische-data/wijk-en-buurtkaart-2016 Statistics Netherlands, District and neighborhoods map 2012. Accessible from: https://www.cbs.nl/nl-nl/dossier/nederland-regionaal/geografische-data/wijk-en-buurtkaart-2012 RIVM, Atlas Living Environment. Accessible from: https://www.atlasleefomgeving.nl/kaarten RIVM, Atlas Natural Capital. Accessible from: https://www.atlasnatuurlijkkapitaal.nl/kaarten

## Abbreviations

SPGH: Self-perceived general health
ML: Machine learning
RF: Random forest
OOB: Out-of-bag
VI: Variable importance
BMI: Body mass index
PM: Particle matter
ALE: Accumulated local effect
ROC: Receiver operating characteristic
PDP: Partial dependance
AUC: Area under the curve
PHM: Public Health Monitor Adults and Elderly of the Community Health Services
RIVM: Dutch National Institute for Public Health and the Environment
