# Supplementary material for: Predicting self-perceived general health status using machine learning: an external exposome study
Source: BMC Public Health. 2023 May 31;23:1027. doi: 10.1186/s12889-023-15962-8 (PMC10230687; doi:10.1186/s12889-023-15962-8)
Supplement: Supplementary file 8 — Additional file 8: Figure S1. Partial dependance plots of a selection of top ranked categorical variables within a random forest model to predict self-perceived general health status. Figure S2. Accumulated local effects plots of a selection of top continuous variables within a random forest model to predict self-perceived general health status. [file 12889_2023_15962_MOESM8_ESM.docx]

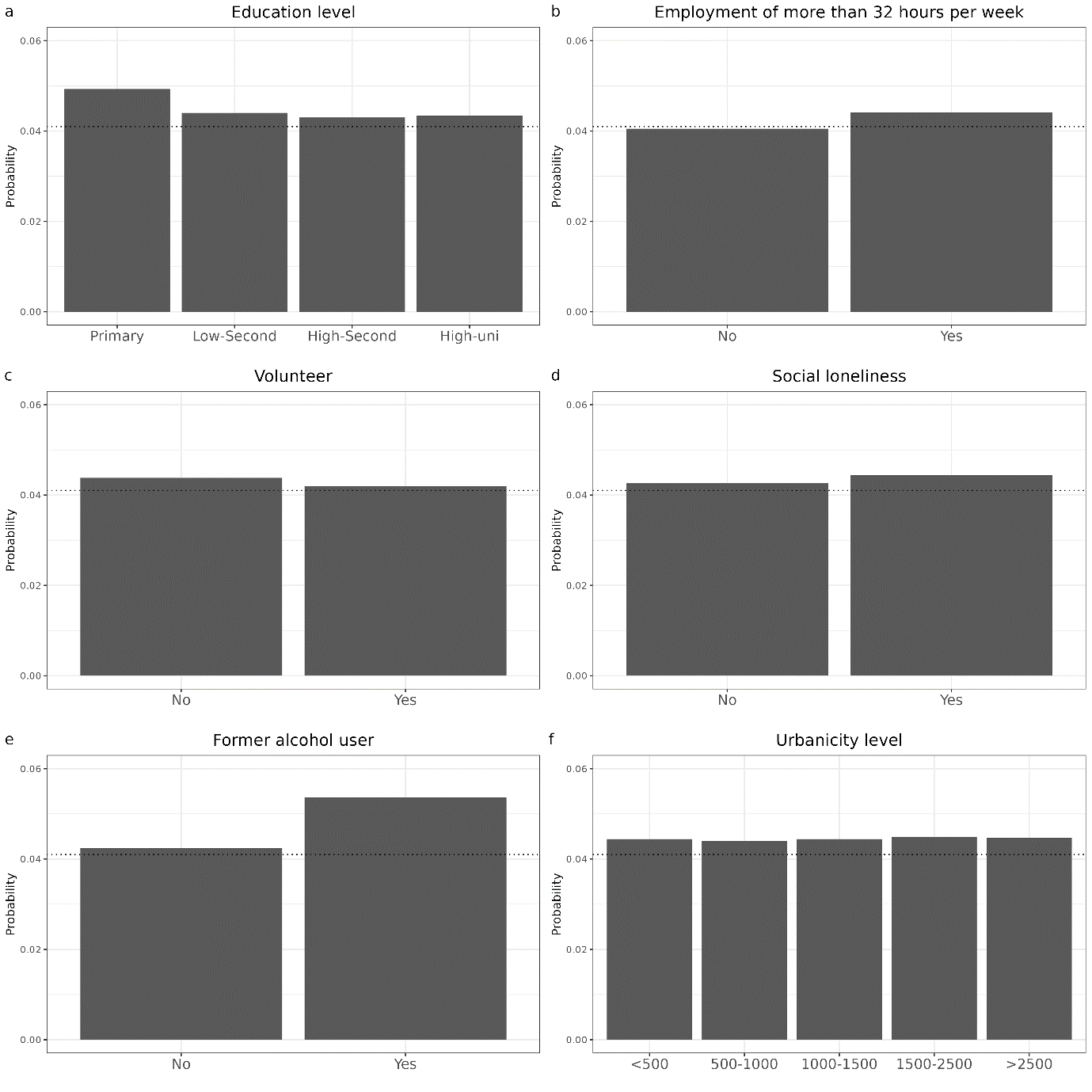


**Figure S1 Partial dependance plots of a selection of top ranked categorical variables within a random forest model to predict self-perceived general health status.**

Legend: The following variables from the complete 2016 random forest model (n = 244,557, variables n = 91) are presented: (**a**) “Education level” (VI rank: 15), (**b**) “Employment of more than 32 hours per week (VI rank: 16), (**c**)“ Volunteer” (VI rank: 17), (**d**) “Emotional loneliness” (VI rank: 18), (**e**) “Former alcohol user” (VI rank: 21) and (**f**) “Urbanity level” (VI rank: 25). Dashed line represents the probability of poor SPGH status in the dataset without taking any exposures into account (0. 041).


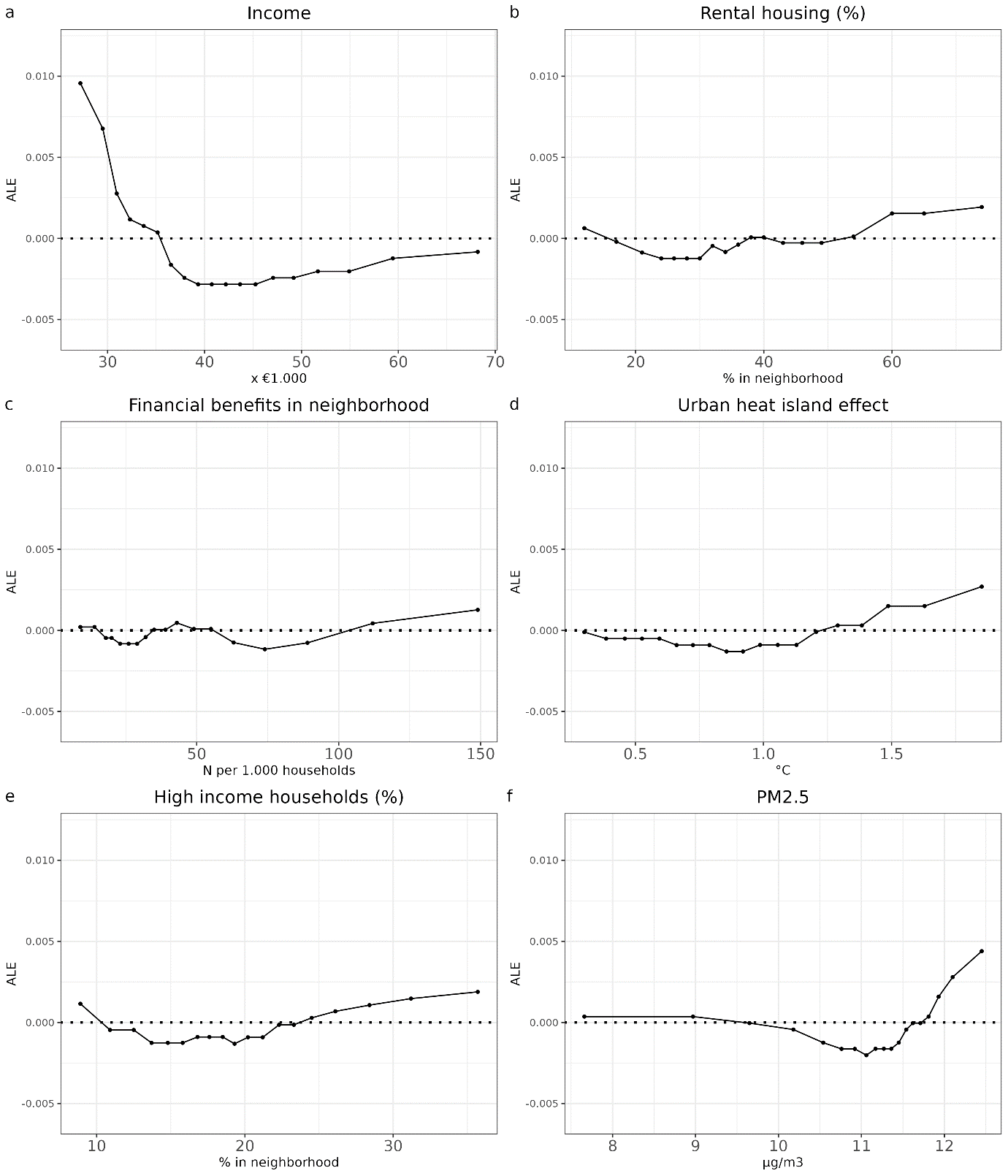


**Figure S2 Accumulated local effects plots of a selection of top continuous variables within a random forest model to predict self-perceived general health status.**

Legend: The following variables from the complete 2016 random forest model (n = 244,557, variables n = 91) are presented: “Income” (**a**, VI rank: 10), “Rental housing (%)” (**b**, VI rank: 24), “Number of financial benefits in neighborhood (n)” (**c**, VI rank: 26), “Urban heat island effect” (**d**, VI rank: 28), “High income households (%)” (**e**, VI rank: 29) and “PM2.5” (**h**, VI rank: 30). Points represent the percentile rank in steps of 5 percentile points, starting from the 5th and ending with 95th percentile of population. The dashed line represents the (normalized) average prediction of the entire RF model.
